# Supplementary material for: Cross-Cultural Adaptation and Validation of the “Brief Scale of Perceived Barriers to Physical Activity for Children”: Analysis of Psychometric Properties
Source: Healthcare (Basel). 2025 Nov 20;13(22):2991. doi: 10.3390/healthcare13222991 (PMC12652913; doi:10.3390/healthcare13222991)
Supplement: Supplementary file 1 [file healthcare-13-02991-s001.zip › healthcare-3928575-supplementary.pdf]

File S1: ESCALA BREVE DE PERCEPCIÓN DE BARRERAS PARA LA PRÁCTICA DE ACTIVIDAD FÍSICA.

No participo en alguna actividad física o deporte fuera del colegio porque…	*Totalmente en desacuerdo*	*En desacuerdo*	*Neutral*	*De acuerdo*	*Totalmente de acuerdo*
1. Porque tengo muchos deberes	1	2	3	4	5
2. Porque los días de entrenamiento / actividades deportivas no me vienen bien	1	2	3	4	5
3. Porque mis amigos/as no practican deporte/s	1	2	3	4	5
4. Porque no estoy en buena forma física	1	2	3	4	5
5. Porque no me interesa el deporte	1	2	3	4	5
6. Porque me da vergüenza mi cuerpo cuando practico deporte	1	2	3	4	5
7. Porque no disfruto con el deporte	1	2	3	4	5
8. Soy peor que los/las demás en el deporte	1	2	3	4	5
9. Porque nadie me dice de hacer deporte	1	2	3	4	5
10. Porque no hay deportes que me gusten	1	2	3	4	5
11. Porque tengo miedo a que se rían de mi	1	2	3	4	5
12. Porque pienso que ya hago suficiente deporte en las clases de educación física	1	2	3	4	5
